# Low- versus Mid-frequency Raman Spectroscopy for *in Situ* Analysis of Crystallization in Slurries

**DOI:** 10.1021/acs.molpharmaceut.2c00126

**Published:** 2022-05-03

**Authors:** Jaana Koskela, Joshua J. Sutton, Tiina Lipiäinen, Keith C. Gordon, Clare J. Strachan, Sara J. Fraser-Miller

**Affiliations:** ∥ Drug Research Program, Division of Pharmaceutical Chemistry and Technology, Faculty of Pharmacy, University of Helsinki, Helsinki FI-00014, Finland; ‡ The Dodd-Walls Centre for Photonic and Quantum Technologies, Department of Chemistry, University of Otago, Dunedin 9054, New Zealand

**Keywords:** Crystallization, amorphous, suspension, low-frequency Raman spectroscopy, *in situ* monitoring, indomethacin

## Abstract

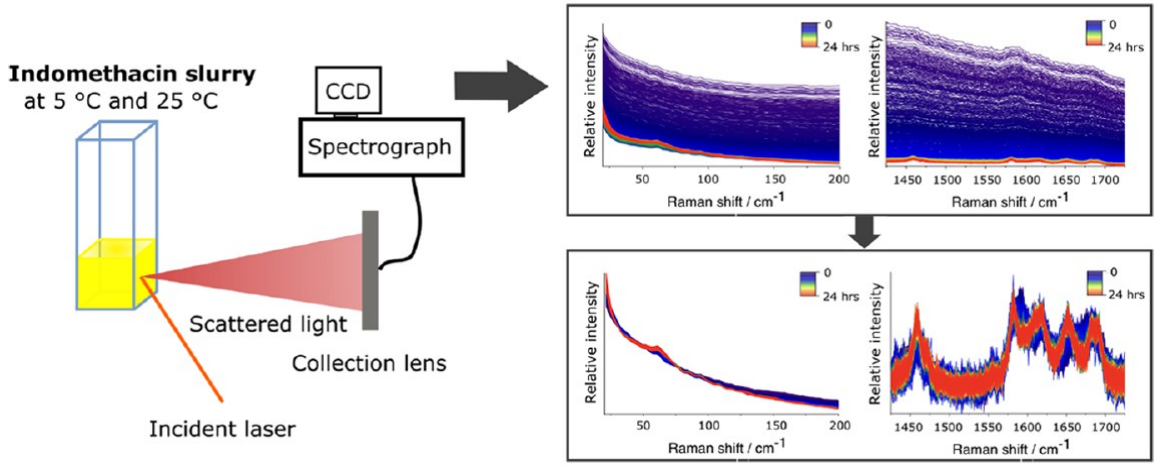

Slurry studies are useful for exhaustive polymorph and solid-state stability screening of drug compounds. Raman spectroscopy is convenient for monitoring crystallization in such slurries, as the measurements can be performed *in situ* even in aqueous environments. While the mid-frequency region (400–4000 cm^–1^) is dominated by intramolecular vibrations and has traditionally been used for such studies, the low-frequency spectral region (<200 cm^–1^) probes solid-state related lattice vibrations and is potentially more valuable for understanding subtle and/or complex crystallization behavior. The aim of the study was to investigate low-frequency Raman spectroscopy for *in situ* monitoring of crystallization of an amorphous pharmaceutical in slurries for the first time and directly compare the results with those simultaneously obtained with mid-frequency Raman spectroscopy. Amorphous indomethacin (IND) slurries were prepared at pH 1.2 and continuously monitored *in situ* at 5 and 25 °C with both low- and mid-frequency Raman spectroscopy. At 25 °C, both spectral regions profiled amorphous IND in slurries as converting directly from the amorphous form toward the α crystalline form. In contrast, at 5 °C, principal component analysis revealed a divergence in the detected conversion profiles: the mid-frequency Raman suggested a direct conversion to the α crystalline form, but the low-frequency region showed additional transition points. These were attributed to the appearance of minor amounts of the ε-form. The additional solid-state sensitivity of the low-frequency region was attributed to the better signal-to-noise ratio and more consistent spectra in this region. Finally, the low-frequency Raman spectrum of the ε-form of IND is reported for the first time.

## Introduction

Crystallization is a crucial process in pharmaceutical development with the stability and the solubility of the active pharmaceutical ingredient largely dictated by the solid-state properties.^1^ During the early phases of drug development, high-throughput crystallization screening is carried out to find the most favorable solid-state form for a new drug candidate.^2^

Real time monitoring can provide a more complete understanding of crystallization. Multiple analytical tools can be used for this purpose, but for *in situ* monitoring, Raman spectroscopy is especially convenient. The technique gives information on both the chemical and the solid-state properties of the compound, and the measurements are fast and nondestructive and can be performed even in aqueous environments.^3,4^ Spectral changes associated with solid-state transformations can, however, be subtle and difficult to detect in the conventional mid-frequency Raman (MFR) spectral region (400–4000 cm^–1^). This is because these signals originate from intramolecular vibrations and may be less sensitive to the subtle changes in crystal structure that are rather mute to the amorphous form. Low-frequency Raman (LFR) spectroscopy (<200 cm^–1^) may be a much more incisive probe of the crystalline state because many of the transitions at these low frequencies are associated with intermolecular vibrations or phonon modes. These modes are intrinsically sensitive to the nature of the crystal state (the polymorph).^5^ The LFR spectra are similar in their information content to that of THz spectroscopy,^6,7^ which has found wide application in polymorph identification.^8^ For example, quantitative solid-state analysis of pharmaceutical ternary powder mixtures gave better results in terms of lower errors of prediction of the models in the LFR region when compared to the MFR region.^9^ Even in the amorphous form, the LFR spectrum gives distinct broad spectral features, sometimes termed the Boson peak, arising from the excess of the density of states relative to the Debye level.^10−12^ These can be used to interpret important properties, such as molecular mobility.^13^

In slurries, where the drug is dispersed in an aqueous external phase, solid-state transformations are accelerated as the conversion can occur directly from the solid or via solution, making slurry studies useful for exhaustive polymorph and stability screening. As an example, Surwase et al.^14^ examined the crystallization of amorphous indomethacin (IND) in slurries with aqueous media and different pH values and temperatures. At 5 °C and pH 1.2, attenuated total reflection (ATR) FTIR analysis revealed the amorphous drug sequentially crystallized to three new polymorphic forms, ε, η, and ζ, which had not been previously published, before the α-form finally emerged. However, at 25 °C, the slurries converted directly to the α-form.

Crystallization of amorphous IND has been studied with multiple techniques and in different conditions. The amorphous form of IND has a glass transition temperature of around 45 °C, and the recrystallization product depends largely on the environment to which the solid is exposed. For example, during storage at low humidity, crystallization leads predominantly to the γ-form, whereas at higher humidity, the α-form crystallizes.^15^ Multimodal nonlinear optical imaging has been used to image the crystallization of amorphous IND on tablet surfaces at very early stages.^15^ The same analytical technique has also been used to assess the effect of solution-mediated crystallization of IND on tablet surfaces during intrinsic dissolution studies.^16^ The study showcased that after 15 min of dissolution in phosphate buffer (pH 6.8) at 37 °C up to four different polymorphic forms occurred simultaneously. *In situ* Raman microscopy and polarized light microscopy have also been used to examine solution-mediated crystallization during intrinsic dissolution testing.^17^

Crystallization of amorphous indomethacin to the γ-form in solid samples has been monitored with both LFR and MFR spectroscopy.^18,19^ Hédoux et al.^19^ examined the crystallization of amorphous IND prepared with two different methods: quench cooling and grinding at −196.15 °C. The method used to compare the two spectral regions explored the first visible traces of crystallization in the LFR and the MFR spectra. A conclusion was that the LFR (5–250 cm^–1^) was more sensitive for the detection of the first traces of crystallization when compared to the C=O stretching region (1500–1750 cm^–1^). Larkin et al.^18^ also compared the two spectral regions, LFR (8–200 cm^–1^) and MFR (1550–1750 cm^–1^), to monitor crystallization of amorphous IND (prepared by quench-cooling the melt) over 8 days. Raman band height ratios of the amorphous and crystalline signals were examined. A comparison of the intensity of crystalline bands between the LFR and MFR regions at 32/1621 cm^–1^ showed that, at low crystallinity, the contribution of the MFR peak was greater when compared to that of the LFR peak. In contrast, at 32/1699 cm^–1^, the peak in the MFR region exhibited less Raman intensity early on.

LFR spectroscopy has been used to quantitatively analyze ternary powder blends comprising different polymorphic forms of piroxicam.^9^ Furthermore, transmission LFR spectroscopy has been used to quantify binary mixtures of carbamazepine polymorphs compacted into tablets.^20^ Larkin et al.^18^ showed that LFR spectroscopy can be used to detect different concentrations of IND in suspensions. In addition, Salim et al.^3,4^ demonstrated that LFR spectroscopy can be used to monitor *in situ* solubilization of suspended ferroquine in milk during digestion. Inoue et al.^21^ have also monitored solid-state transformations of carbamazepine *in situ* with LFR spectroscopy involving a sampling probe. Form III was observed to transform into form I upon heating the solid. The dissolution of form I into warm ethanol followed by precipitation of form III upon cooling the solution was also detected. In addition, dihydrate formation could be monitored when form III was dispersed in different ratios of ethanol–water mixtures, and the transformation kinetics were determined using multivariate curve resolution.

Despite the multiple studies carried out to utilize LFR spectroscopy as an *in situ* monitoring tool,^22−24^ the solid-state changes of an amorphous pharmaceutical in slurries have not yet been probed. The inherently longer-range order requirement for defined vibrational modes in the LFR range when compared with MFR warrants further comparison of the two regions with respect to early stage crystallization detection. The aim of this study was to compare LFR and MFR spectroscopy for *in situ* monitoring of crystallization of an amorphous pharmaceutical in slurries. The crystallization of amorphous IND in slurries at two temperatures, 5 and 25 °C, was monitored *in situ* with LFR and MFR spectroscopy. The resulting spectra were analyzed using principal component analysis (PCA) and partial least-squares discriminant analysis (PLS-DA).

## Experimental Section

### Solid-State Form Preparation

Indomethacin (IND) (Sigma-Aldrich, Saint Louis, USA, and Hawkins Pharmaceutical group, Minnesota, USA) was used as a model drug, and the bulk material was in the γ-form. The amorphous form was prepared from the γ-form by quench cooling the molten drug on a heat sink placed on ice. The resulting glass was placed in a desiccator over dry silica gel for 30 min and then ground into a powder with a mortar and pestle. The α-form was prepared by dissolving the γ-form in ethanol at 80 °C and thereafter adding Milli-Q water at room temperature to initiate precipitation.^25^ The δ-form was obtained by desolvation of IND methanolate.^26^ The ε-form was obtained from an amorphous IND suspension prepared with pH 6.8 phosphate buffer.^27^ The solid-state forms were verified by Raman spectroscopy, Fourier transform infrared spectroscopy and X-ray powder diffraction and are designated as the 0 timepoint for offline measurements.

### Sample Preparation

For the off-line measurements, the amorphous IND slurries (30 mg/mL) were prepared by adding 10 mL of 0.063 M aqueous HCl solution (pH 1.2) in a 20 mL scintillation vial. The solids were dispersed in the media by stirring the sample in a closed vial at a high speed for 15 s. Thereafter, the vial was kept in an oil bath at 5 or 25 °C under continuous stirring at 500 rpm with a magnetic stirring bar for 24 h. Samples were taken from the slurries at different time points for spectroscopic analysis including Fourier-transform (FT) Raman spectroscopy, attenuated total reflection Fourier transform infrared (ATR-FTIR) spectroscopy, and LFR spectroscopy. Samples (500 μL) taken at 30, 60, 120, 240, 480, and 1440 min were centrifuged, and the wet solid was analyzed. Slurries were prepared and the samples, analyzed in triplicate.

For the *in situ* analysis, amorphous IND slurries (30 mg/mL) were prepared by adding 550 μL of 0.063 M aqueous HCl solution (pH 1.2) into a quartz cuvette (10 × 10 mm). To evenly disperse the solid, the suspension was mixed with a Vortex touch mixer for 15 s. After mixing, the cuvette was placed in a temperature-controlled cuvette holder and *in situ* monitoring with Raman spectroscopy was initiated. The slurry was kept at 5 or 25 °C under continuous mixing with a magnetic stirring bar. Vortex mixing was repeated at 5, 15, 30, and 60 min time points to avoid aggregation of the solids.

### Scanning Electron Microscopy

The morphology of the particles in 25 °C slurries at different time points was examined with scanning electron microscopy (SEM) (Quanta FEG 250, FEI Company, Hillsboro, OR, USA). Samples (400 μL) taken at 3, 60, 120, 240, and 1440 min were centrifuged, and the supernatant was removed. The remaining wet solid was dried under vacuum for 30 min at room temperature. The dried solid was gently broken up with a spatula and placed on carbon tape attached to an aluminum stub. Excess particles were removed with compressed air. Samples were coated with gold using a SCD 005 Cool Sputter Coater (Bal-Tec AG, Balzers, Liechtenstein) with a current of 20 mA for 300 s.

### FT-Raman Spectroscopy

The FT-Raman spectrometer consisted of a multiRam FT-Raman spectrometer (Bruker Optik, Ettlingen, Germany), 1064 nm Nd:YAG laser, and a D 418 Ge detector. Spectra were collected using the defocusing lens to give a 2 mm diameter spot size, a laser power of 150 mW, and 4 cm^–1^ resolution with each spectrum having an average of 128 scans. Each off-line sample was measured in triplicate.

### FTIR Spectroscopy

ATR-FTIR measurements were carried out using a purged Vertex70 FTIR spectrometer (Bruker Optics, Ettlingen, Germany) with a GladiATR diamond ATR accessory (Pike Technologies, Madison, WI, USA). A background was acquired before each sample spectrum, and each spectrum was the average of 256 scans collected over 50–4000 cm^–1^ at a 4 cm^–1^ spectral resolution. Each off-line sample was measured in triplicate.

### Low-Frequency Raman Spectroscopy

*In situ* and off-line analysis was carried out with a purpose-built Raman setup^4,9,23,28^ equipped with a 785 nm excitation laser (Ondax, Monrovia, CA, USA). Before the sample was irradiated with a backscattering geometry, amplified spontaneous emission was removed by using BragGrate bandpass filters (OptiGrate, Oviedo, FL, USA). The focal spot diameter was approximately 500 μm. Light scattered from the slurry (*in situ*) or solid placed in a divot (off-line) was collected and filtered through a set of volume Bragg gratings (OptiGrate, Oviedo, FL, USA) and then focused into an LS 785 spectrograph (Princeton Instruments, NJ, USA) via fiber optics, which dispersed the scattered light onto a Pixis 100 BR CCD detector (Princeton Instruments, Trenton, NJ, USA). The spectral region was recorded from −345 to 2055 cm^–1^ with a resolution of 5–7 cm^–1^. This means the low-frequency (LF) and mid-frequency (MF) spectral data were collected simultaneously, allowing for a direct comparison between the two regions. *In situ* measurements were conducted for 24 h by collecting one spectrum per minute (1 s integration × 60 coadditions). The off-line measurements were acquired with a 0.1 s integration time and 1200 coadditions to have a total measurement time of approximately 2 min per spectrum with each replicate time point measured in triplicate. The *in situ* samples were prepared and measurements, carried out in duplicate; the off-line samples were assessed in triplicate.

### Spectral Preprocessing and Multivariate Analysis

The 785 nm Raman spectra (*in situ* and off-line) were preprocessed to remove focused based differences between runs. For the LFR, a linear baseline correction (−200 to 200 cm^–1^; anti-Stokes was included to minimize the alteration of the virtual density of state (VDOS) signals) followed by standard normal variate (SNV) normalization (20 to 200 cm^–1^) was used. For the MFR, a linear baseline correction followed by SNV was carried out on the 1425 to 1725 cm^–1^ spectral window. The FT-Raman spectra (off-line) underwent the same preprocessing as the 785 nm MFR; linear baseline correction followed by SNV over the spectral region 1425 to 1725 cm^–1^. The FTIR spectra were preprocessed with SNV over the spectral window of 1500 to 970 cm^–1^.

PCAs of the 5 °C (*n* = 2) and 25 °C (*n* = 2) *in situ* runs were carried out together with the LFR (20–200 cm^–1^) and MFR (1425–1725 cm^–1^) spectral windows analyzed separately. PCA was calculated on the preprocessed spectra with the NIPALS algorithm and random cross validation with 20 segments.

The 25 °C data underwent PLS-DA to explore the kinetics of forming α-IND from amorphous IND. For the *in situ* data, the first three spectra from each run were given the dummy variable “0” and the final three spectra from the two runs were designated “1”. For the off-line data, the 0 min time point was designated “0” and the 1440 min time point samples were designated “1”. A PLS model was generated for each technique with the kernel PLS algorithm and full cross validation. All other spectra were then projected onto the models. The spectral preprocessing, PCA, and PLS-DA analyses were carried out with Unscrambler X (version 10.4, Camo Software AS, Oslo, Norway).

## Results and Discussion

### Particle Morphology

Visual inspection with off-line SEM of the particles from the slurries at 25 °C was carried out to gain information about the morphological changes of the suspended particles over time. Indomethacin solid-state forms have distinct differences in their morphologies: the γ-form and amorphous form (prepared by cooling the melt and then grinding in this study) have prismatic shaped particles whereas the α-form has needle-shaped particles.^15,29^

**Figure 1:**
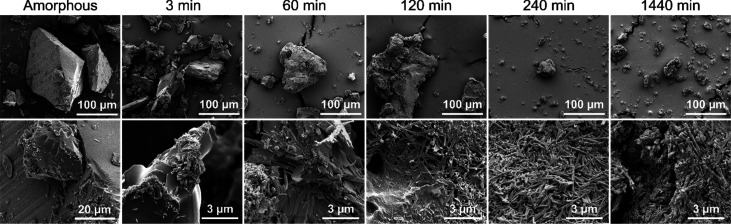
SEM micrographs of amorphous IND particles and samples taken from slurry at 3, 60, 120, 240, and 1440 min and 25 °C. Magnifications: top row 1000× and bottom row 5000× and 30 000× for amorphous and slurry samples, respectively.

The SEM micrographs of the samples taken at different time points suggest crystallization to the α-form (Figure 1). Around the beginning (3 min), the surfaces of the particles were mostly smooth and largely resembled the amorphous particles not exposed to the medium. Minuscule regions with small needle-like shapes were, however, seen on the particles at 3 min. Small fragments could also be seen on the amorphous particles, but the shape of these fragments was somewhat random. At 60 min, the surfaces were largely covered with needle-shaped structures. The surface morphology continued to change over the 60 to 1440 min time period. The coverage became more complete and thicker, and the surface crystals at 1440 min were slightly thicker with a lower aspect ratio than at 240 min. The SEM micrographs can, however, only give an estimate of the behavior of the slurries, as the sample preparation could potentially cause additional solid-state changes in the samples. To explore the solid-state nature of the morphological changes, *in situ* low- and mid-frequency Raman spectroscopy was used.

### *In Situ* Raman Spectroscopy Analysis

#### Visual Inspection of LFR and MFR Spectra

**Figure 2:**
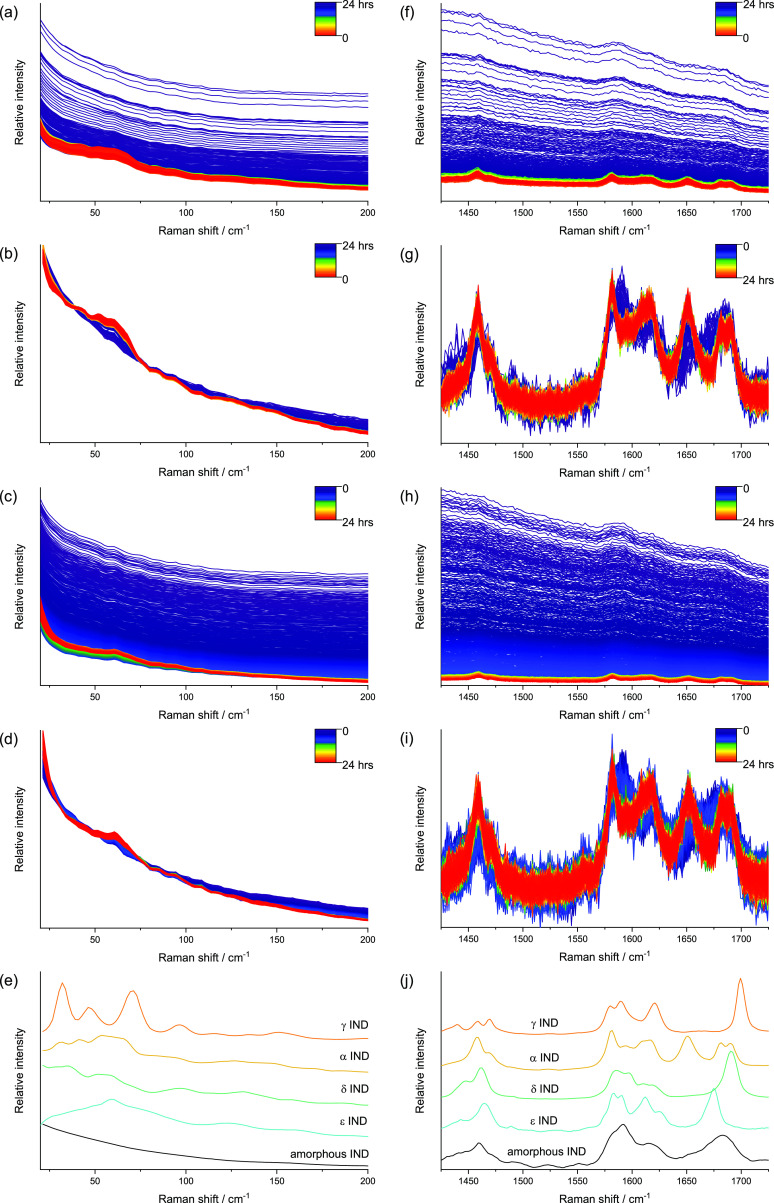
Example Raman spectra from the 25 and 5 °C runs. (a) 25 °C LFR raw data, (b) 25 °C LFR preprocessed data, (c) 5 °C LFR raw data, (d) 5 °C LFR preprocessed data, (e) reference LFR spectra of indomethacin polymorphs, (f) 25 °C MFR raw data, (g) 25 °C MFR preprocessed data, (h) 5 °C MFR raw data, (i) 5 °C MFR preprocessed data, and (j) reference MFR spectra of indomethacin polymorphs.

The raw and preprocessed Raman spectra from representative *in situ* runs, collected from both the LFR and MFR, are presented in Figure 2. Each spectrum represents an average signal from the solution and dissolved and solid indomethacin, with the last one dominating the signal. Prior to preprocessing, the Raman signal intensity in the LFR was substantially higher than that in the MFR (Figure S1). This intensity difference between the two spectral regions was discussed earlier by Lipiäinen et al.^9^ The enhanced signal intensity from the low-frequency region is in part a consequence of the Raman scattering dependence on ν;^4^ as the low-frequency scattered photons are at shorter wavelengths, they intrinsically scatter more effectively.^5^

In the MFR, the bands in the 1600–1725 cm^–1^ region correspond to those in previously published Raman spectra of the amorphous and α-forms of IND (Figure 2g,j).^30−32^ The peaks arising from both the benzoyl and acid C=O stretching are frequently used to differentiate the different solid-state forms of IND. At the beginning of the *in situ* monitoring, for both the 5 and 25 °C runs, a broad peak at around 1680 cm^–1^ due to benzoyl C=O stretching in amorphous IND was present (Figure 2g,i).^30−32^ At 1440 min, the broad peak had disappeared at both temperatures and was replaced by three narrower peaks corresponding to the α-form of IND. The peak at 1691 cm^–1^ arises from non-hydrogen bonded benzoyl C=O stretching,^30,32^ while those around 1683 and 1652 cm^–1^ represent hydrogen bonded carbonyl acid C=O stretching of the α-form.^30,32^

The first Raman spectrum recorded during the continuous monitoring for both the 5 and 25 °C runs exhibited no distinct spectral peaks in the LFR, and the spectrum corresponds to the amorphous IND spectrum superimposed over the aqueous media (Figure 2a–e). Spectra without the peaks in the LFR are characteristic of the amorphous form, which shows typical broad features associated with the VDOS.^32,33^

At the end of the monitoring (1440 min), two subtle phonon peaks appear in the LFR at around 42 and 53 cm^–1^ and one broader peak is around 60 cm^–1^ (Figure 2b,d). These peaks correspond with previously published LFR spectra of the α-form of IND.^19,32,34^ However, in contrast to the reference spectra of the α-form, the LFR had an elevated baseline at the end of the monitoring. The baseline effect is caused by the contribution of the aqueous media in which the IND is suspended. The vibrational nature of the phonon modes associated with the α-form of IND were elucidated by Ruggiero et al.^34^ with the aid of theoretical solid-state density functional theory and *ab initio* molecular dynamics calculations. Briefly, most of the peaks below approximately 44 cm^–1^ were attributed to the conformationally rigid hindered rotational motions, whereas the phonon modes with higher frequencies were mostly attributed to the internal torsion-type motions.

When no preprocessing was applied, the baseline intensity decreased substantially during the first 120 min in both regions and at both temperatures (Figure 2a,c,f,h). This may be due to non solid-state related factors, such as how well the particles are dispersed into the medium. However, the change in the shape of the baseline observed in MFR indicates that photoluminescence of the amorphous form plays a key role in the baseline shift.

In the MFR, the 25 °C runs featured α-IND peaks beginning to appear within 30 min (seen most easily with the preprocessed data, Figure 2g). The intensity of these benzoyl and carbonyl acid C=O stretching peaks increased up to approximately 200 min, after which no substantial changes were visually apparent. The loss of the broad emissive signal in the raw data also occurs on a similar time frame, Figure 2f. Overall, the spectra recorded in the MFR at 25 °C suggest a direct conversion of the amorphous form toward the α-form without any intermediate forms. This is consistent with solution-mediated direct conversion of the suspended amorphous form to the α-form at 25 °C and pH 1.2 observed by Surwase et al.^14^ The 5 °C runs appeared to have similar features growing in (Figure 2i), albeit more slowly with no substantial changes after approximately 700 min.

In the LFR, the 25 °C runs also show signs of crystallization within 30 min (Figure 2b). A single peak started to evolve at around 61 cm^–1^, attributed to internal torsional-type molecular motion.^34^ The peak can be associated with more than one IND solid-state form: the most intense peaks for both the α- and ε-forms appear around this region (Figure 2b). The α-form has a broad band from around 46 to 75 cm^–1^ with two peak maxima at around 54 and 64 cm^–1^. The ε-form, of which the LFR spectrum is published here for the first time, has a wide almost symmetrical peak from around 23 to 100 cm^–1^. The wide peak sharpens between 54 and 66 cm^–1^ with the apex at around 60 cm^–1^. More peaks associated with the α-form appeared later on: within 45 min, a subtle peak appeared at approximately 52 cm^–1^, and after 120 min another one appeared at approximately 42 cm^–1^. These peaks are due to long/short-range order.^34^ The intensity of all the peaks grew over time, and after 400 min, no substantial intensity changes were observed by visual inspection. The 5 °C runs exhibit more subtle LFR changes; however, the spectral profiles appear to more closely resemble the ε-form (Figure 2d).

While visual inspection provided an overview of the transformations potentially taking place, the analysis of a large set of spectral data is time-consuming, and subtle changes in the spectra might be overlooked, especially from a kinetic perspective. Thus, multivariate analysis was used to get more insight into the pathway and kinetics of the crystallization.

#### Principal Component Analysis

**Figure 3:**
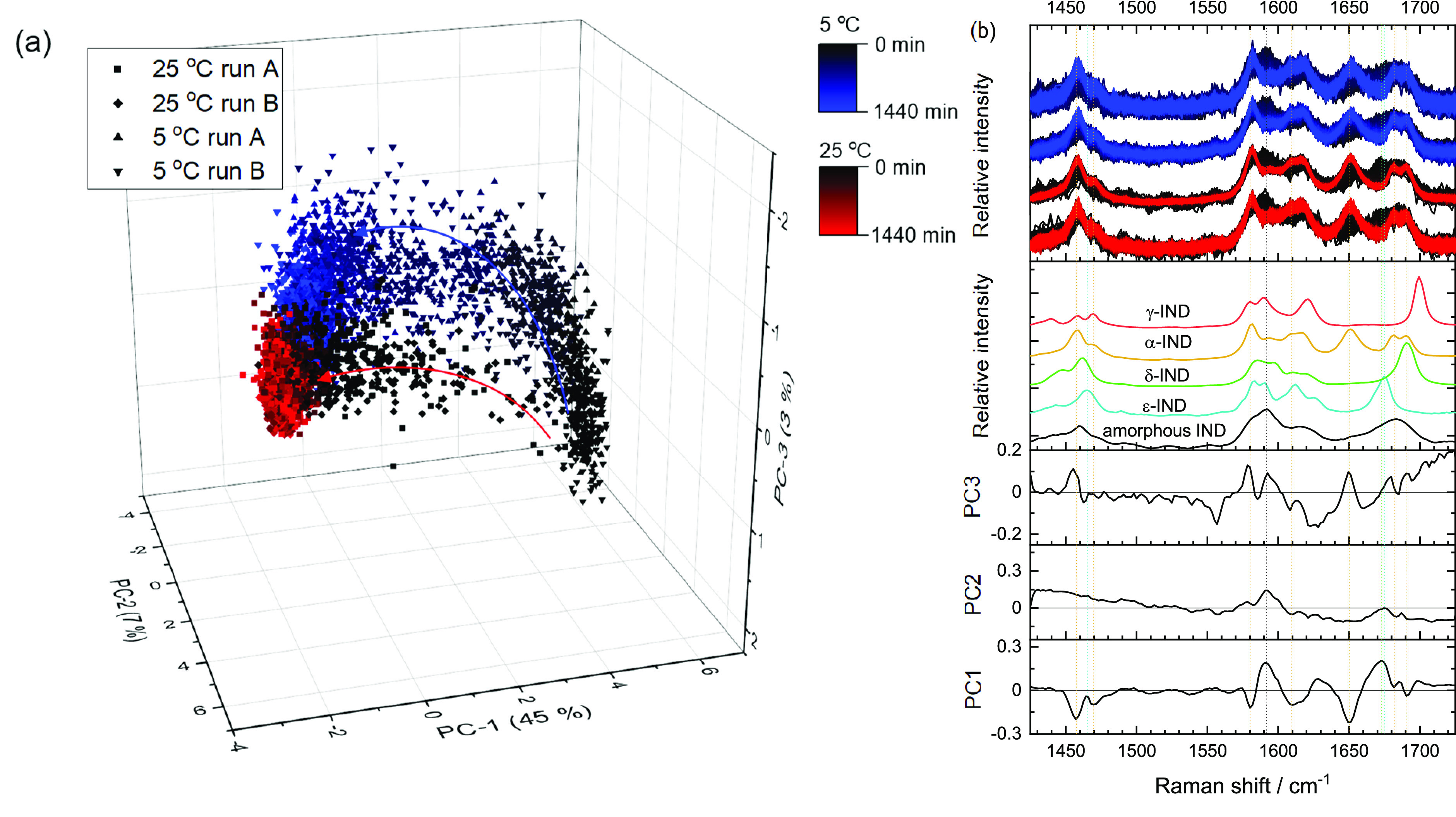
Duplicate runs of the 5 and 25 °C samples with the MFR spectral region analyzed with PCA. (a) Scores plot for the first 3 PCs, accounting for 55% of the explained spectral variance and (b) the associated loadings plots and reference spectra in comparison to the spectra generated during the replicate runs.

PCA was carried out on the LFR and MFR regions for the duplicate 5 and 25 °C runs to get an initial overview of the spectral trends and observed conversion profiles. With the MFR, all runs (5 and 25 °C) moved directly from positive PC1 (amorphous IND signals) to negative PC1 (α-IND signals) score space with a smooth transition in PCs 1, 2, and 3 (no obvious sharp turning points, Figure 3a). Slight differences between the 5 and 25 °C trajectories were observed with the 5 °C tending to have more negative PC3 space and 25 °C tending to have more positive PC3 space. There were observable differences in the movement of samples with time in PC space between the 5 and 25 °C runs. When looking at PC1 versus time, it is apparent that the 25 °C sample spectra reached equilibrium after 200 min and the 5 °C run spectra did not reach equilibrium until approximately 600 min (Figure S2).

**Figure 4:**
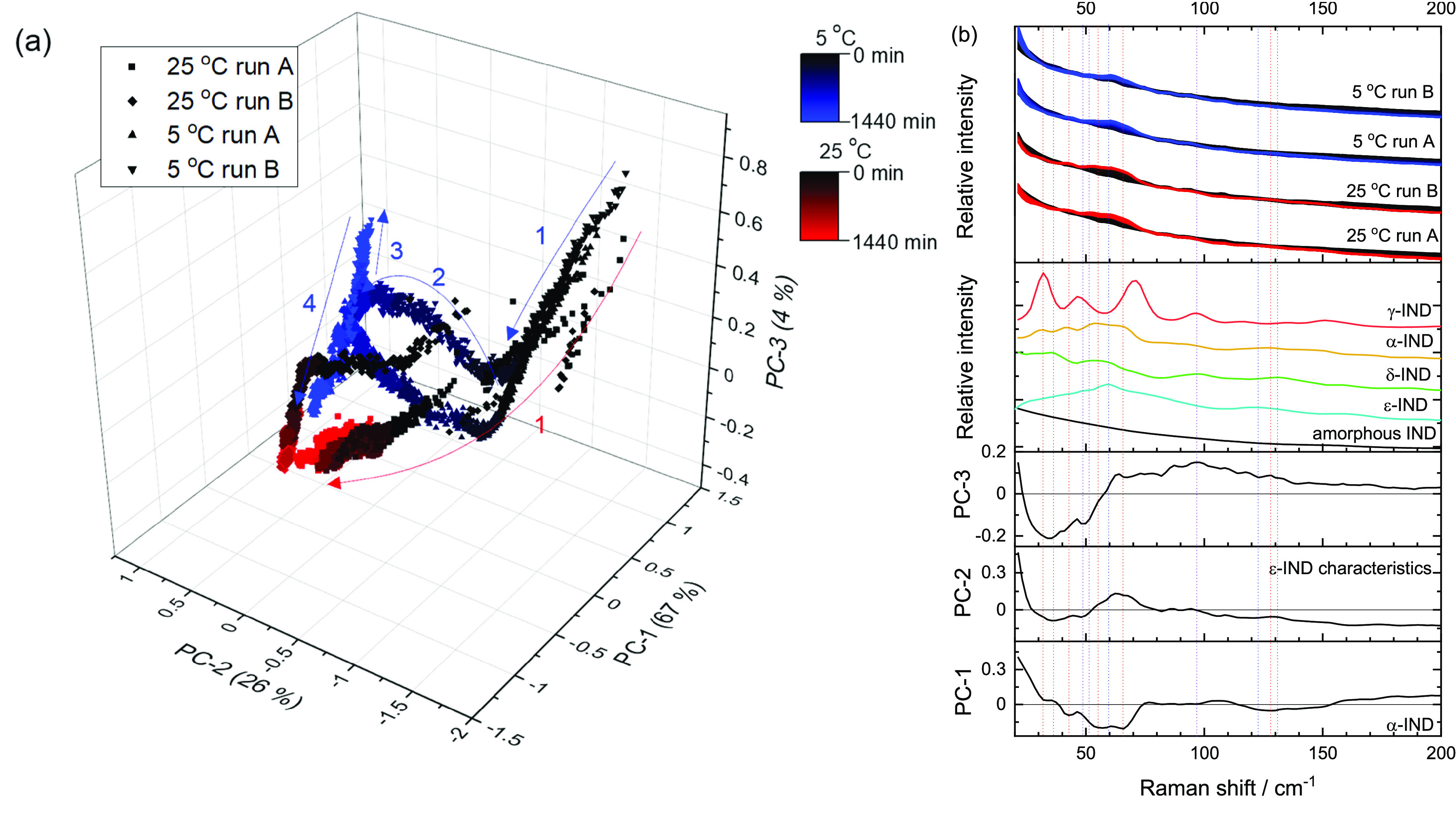
Duplicate runs of the 5 and 25 °C samples with the LF spectral region analyzed with PCA. (a) Scores plot for the first 3 PCs, accounting for 97% of the explained spectral variance and (b) the associated loading plots and reference spectra in comparison to the spectra generated during the replicate runs. The numbers and arrows in (a) indicate the movement of samples with time and the changing direction of this sample movement in the PC score space.

The LFR gave a similar trend for the 25 °C sample with the spectra appearing to smoothly move from amorphous (positive PC1, negative PC2, and positive PC3) to α-IND (negative PC1 and neutral PC2 and PC3) in PC scores space (Figure 4). The 5 °C LFR counterparts displayed an interesting series of turning points in PC space, which were observed in the same pattern for both runs. The turning points were observed as follows: the first at ∼280 min, the second at ∼870 min (run A) or ∼600 (run B) min, and the third around 950 min (run A) or 1400 min (run B) after which samples moved toward the α-IND space. Average spectra from the turning points are given in Figure S4. While the times of the turning points differed between runs, the pattern of movement and differences in spectra between the turning points were similar. These turning points are tentatively attributed to transient and minority intermediate metastable ε-polymorph formation. The loadings for the first three PCs appear to describe different features present in different solid-state forms of IND (Figure 4b). On the basis of the movement in PC space, the loading features, and spectra observed at these turning points, the following set of transitions appear to be occurring: (1) loss of amorphous signals and a change in shape below 40 cm^–1^ with a hint of ε-IND growing in at 62 cm^–1^, (2) increased signals from ε- and α-IND, (3) changes at 30 cm^–1^ and below (possibly indicating changes in particle size/length scales changing the optical/scattering properties of the sample), and (4) growth of α-IND signals.

The direct transition from amorphous to α at 25 °C and pH 1.2 is consistent with the observation of Surwase et al.;^14^ while the observations diverge at 5 °C, Surwase et al. observed the following transitions: amorphous to ε to ζ to η to α. There were, however, several experimental differences between the studies. Sample differences included the rate of cooling the indomethacin melt (over ice vs liquid nitrogen), subsequent grinding of the glass (mortar and pestle vs oscillatory ball mill), and suspension stirring dynamics (cuvette vs vial, volume, stirring speed), all of which may have impacted the crystallization behavior. The measurement procedures also may have had an impact on the observed crystallization behavior with the current study employing online analysis (with Raman), while that of Surwase et al. relied on off-line sampling with sample centrifugation prior to the strongly surface biased ATR-FTIR analysis. In any case, the observed differences serve to highlight the exquisitely sensitive and complex solution-mediated polymorphic behavior of indomethacin upon crystallization at 5 °C with the potential for several forms to coexist during crystallization.

The difference in observed trajectories between the LFR and MFR regions could be due to a couple of factors. One possibility is that the LFR region is more sensitive to the solid-state form due to the intermolecular nature of the vibrations probed. There is also a signal-to-noise (S/N) advantage in the LFR region, meaning the spectra from LFR are potentially more sensitive to subtle changes in sample composition. The signal from the MFR region may just be too noisy to pick up subtle changes in minority polymorph signals. This high relative noise contribution is also expressed in the relative variance explained with PCA with the MF region explaining less spectral variance (3 PCs describe 55% variance) compared with the LF region (3 PCs describe 97% variance) due to the MF data being inherently noisier.

As the 25 °C samples displayed a smooth transition from amorphous to α-IND (as evidenced by both the LFR and MFR regions), it was deemed worthwhile to explore the potential of LFR and MFR to track the kinetics of this transformation. The 5 °C LFR data was not included in this analysis due to the complex nature of the transformation profile (rather than a direct and exclusive transformation to the α-form) complicating the application of supervised multivariate analysis (in this case, partial least-squares discriminant analysis (PLS-DA)).

### Complementary Off-Line Analyses: ATR-FTIR and 785 nm Dispersive Raman

**Figure 5:**
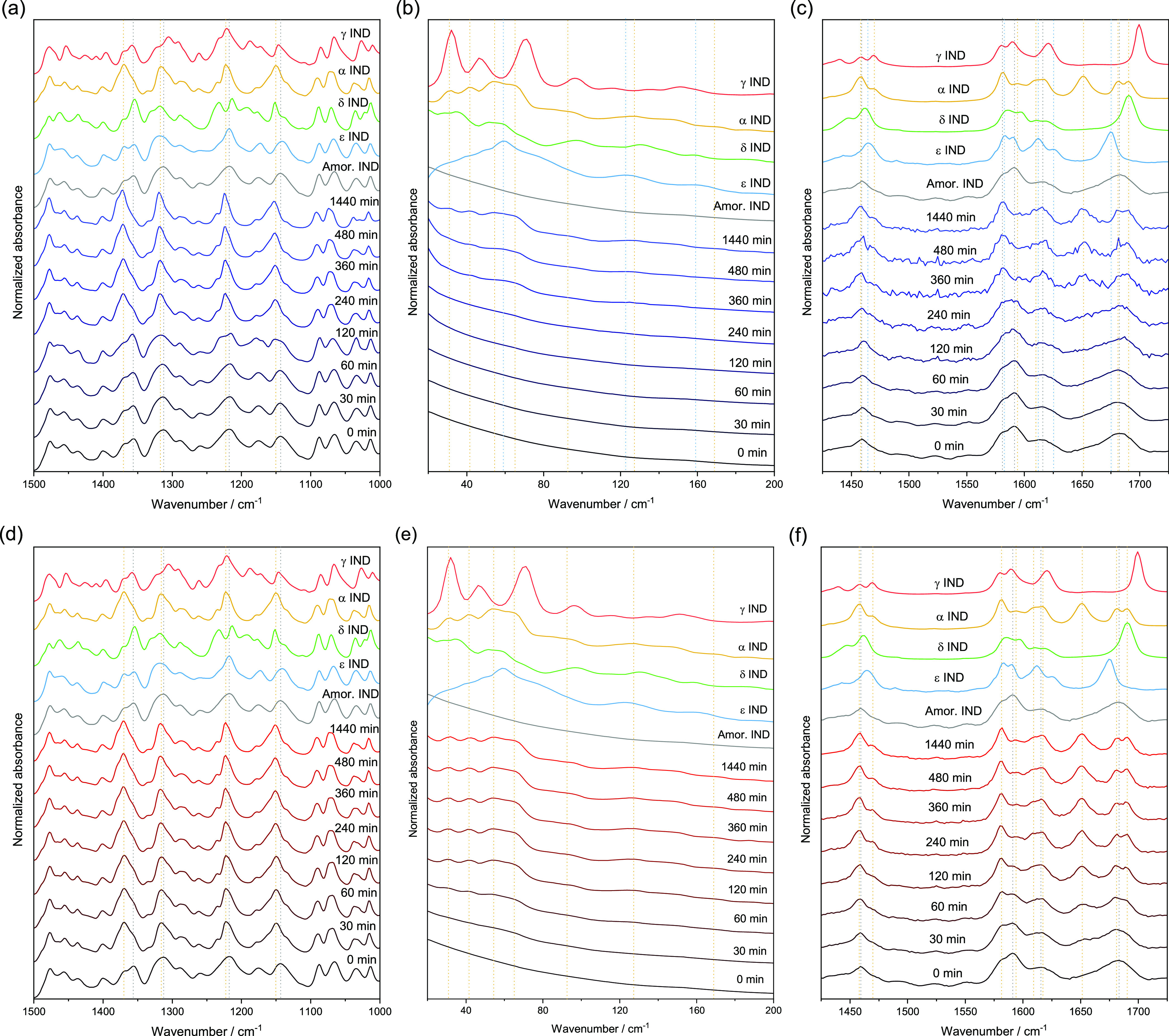
Representative off-line FTIR and Raman measurements collected at 0, 30, 60, 120, 240, 360, 480, and 1440 min: (a) IR 5 °C, (b) off-line LFR 5 °C, (c) off-line MFR 5 °C, (d) ATR-FTIR 25 °C, (e) off-line LFR 25 °C, and (f) off-line MFR 25 °C.

The *in situ* solid-state transformation from amorphous toward the α-form was compared to off-line samples analyzed with ATR-FTIR and 785 nm laser Raman spectroscopy with both LFR and MFR detection (Figure 5). ATR-FTIR spectra of the 5 °C data appeared predominantly amorphous in nature for the 0 to 120 min time points and predominantly the α-form from 240 min onward (Figure 5a). Both the LFR and MFR data exhibit changes across the entire measurement time frame for the 5 °C samples. For the MFR region, the spectra appear amorphous from 0 to 120 min after which the 240–360 min time points exhibited poor S/N ratios, making interpretation difficult. It is worth noting this happened consistently across multiple (*n* = 3) runs where these intermediate time points generated spectra with poor S/N ratios. The latter time points (480 and 1440 min) were consistent with the α-form (Figure 5c). In the LFR region, this poor S/N ratio was not an issue due to the inherent signal advantage in the LFR domain. In the LFR region, the spectra appeared amorphous from 0 to 120 min after which a hint of the ε- or α-form was observed with a broad feature at approximately 60 cm^–1^. The latter time points (360 to 1440 min) showed increasing contribution from the α-form (Figure 5b).

For the 25 °C samples, ATR-FTIR spectra from the 30 min through to the 1440 min time points were consistent with the α-form (Figure 5d). In contrast, the MFR spectra visibly changed up to 120 min (Figure 5f). At 30 min, the spectra had α-form features beginning to grow in, but these peaks were not as sharp and well-defined as at later time points. The LF region also appeared to change on a similar time frame to the MF region with a slow evolution of α-form features to 120 min after which the spectra remain consistent (Figure 5e). Peaks related to the α-form continued to grow and sharpen, and the amorphous features diminish up to 120 min.

### Partial Least-Squares-Discriminant Analysis (PLS-DA) of *in Situ* and Off-Line Measurements

**Figure 6:**
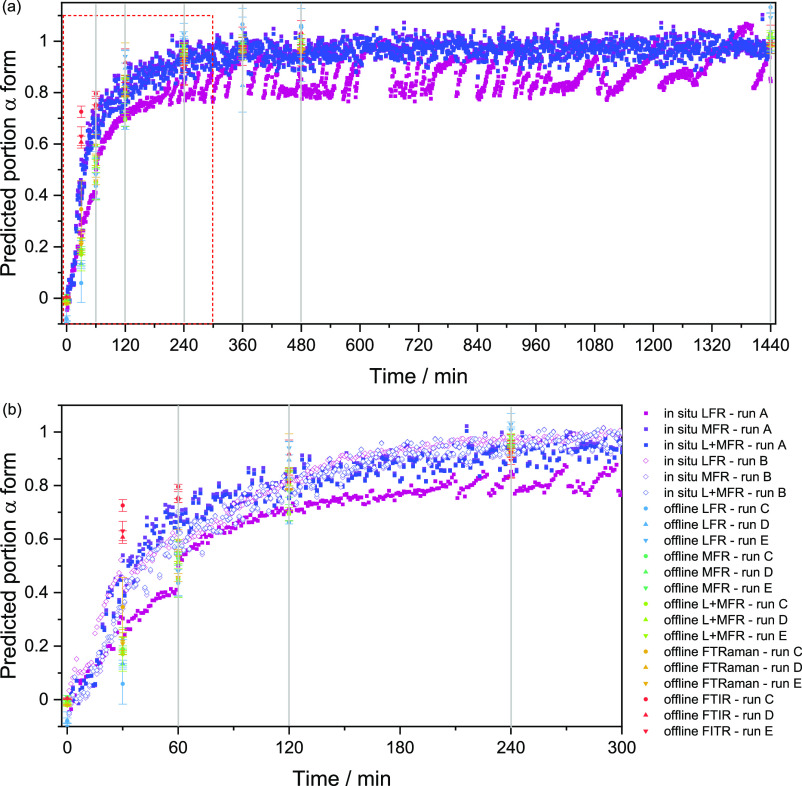
Evolution of α-IND from amorphous IND at 25 °C over time based on PLS-DA projections. (a) The full time period and (b) the first 5 h. These data include both *in situ* runs detected with both LFR and MRF as well as the combined spectral regions along with off-line measurements. Please note the associated regression coefficients for these PLS-DA models are given in Figures S4 to S9.

PLS-DA models were created using the first (designated 0) and last (designated 1) three spectra from the *in situ* Raman runs and the start (0 min) and end (1440 min) points of the off-line runs collected at 25 °C. The regression coefficients for these models are given in Figures S5 to S9. Only one factor was required for each model with the weighted β-coefficient describing the difference between amorphous (negative coefficients consistent with amorphous spectral features) and α-IND (positive coefficients consistent with α-IND spectral features) for each technique. All spectra were then projected through these models to give an approximation of the evolution of α-IND over time (Figure 6). There were slight variations observed between *in situ* runs, in particular the LFR appeared sensitive to particle accumulation on the cuvette side and the dislodging of said particles, which is the attributed source of the variance (oscillations) in the LFR data of run A. To minimize this effect in the future, the focal point of the measurement should be deep within the sample rather than near the cuvette interface. Some avenues to explore to minimize this effect include transmission low-frequency Raman^20,35^ or spatially offset low-frequency Raman^36^ setups, which also have the advantage of probing larger sample volumes.

In comparison to the *in situ* results, the ATR-FTIR data tended to overestimate the proportion of α-IND present and the off-line LFR data appeared to underestimate the proportion present in the early stages. All off-line data gave similar proportions after 120 min (Figure 6). The gray lines in Figure 6 present the time points at which the SEM micrographs were taken (Figure 1). On the basis of the micrographs, the surface of the particles were largely covered with needle-shaped crystals at the 60 min time point. This surface coverage of α-crystals is reflected in the ATR-FTIR spectra and PLS-DA predictions at the 60 min time point, where the predicted α-form concentration reached approximately 80% at 60 min.

The difference between the IR and Raman results can be explained by the sampling depth of the two spectral analysis techniques. ATR-FTIR is a surface-biased method as it only probes approximately 2 μm from the sample surface, while the Raman setup has a substantially deeper sampling depth of approximately 1 mm. The solution-mediated crystallization in suspension begins at the surface of the amorphous particles, and a complete coverage is reached relatively quickly, as evidenced by the SEM analyses (Figure 1). Thus, for the sample at 30 min, the ATR-FTIR spectra indicated a complete transformation, whereas the Raman continued to detect remaining amorphous IND at 60 min (Figure 5).

The off-line analyses only give an estimate of the original sample: the time delay between the sampling and analysis as well as any sample preparation could potentially cause post-extraction solid-state changes in the sample and thus different results. In this study, samples were centrifuged before the analysis, which caused some time delay, and for the SEM, the slurry samples had to be dried. Thus, *in situ* measurements, in addition to being more convenient, can give more precise information related to the crystallization kinetics in slurries over time.

PLS-DA analysis can only give an estimate of the concentration profiles from amorphous to α-form. The discriminant model assumes a complete transformation from the pure amorphous form to pure α-form. However, the crystallization process begins immediately after the solid is exposed to the medium, and the three first spectra recorded and used for building the model can also have some features from the α-form. Also, it is possible that even after 1440 min some particles contain some residual amorphousness buried within the particles, which were not accounted for by the PLS-DA model.

## Conclusions

This study represents the first direct comparison of LFR and MFR for *in situ* analysis of crystallization in amorphous drug slurries. The crystallization behavior of amorphous indomethacin was continuously probed at pH 1.2 with constant stirring at two temperatures: 5 and 25 °C. At 25 °C, both spectral regions suggested a direct solution-mediated crystallization to the α-form. At 5 °C, the observations between the spectral regions diverged: while MFR did not provide evidence of intermediate solid-state forms, PCA analysis of the LFR revealed several transitions, consistent with the appearance of the minority intermediate ε-form. The additional information in the LFR is largely attributed to its better signal-to-noise ratio compared to MFR, which in turn, increases sensitivity to minority and transient crystal forms. Finally, we also present the LFR spectrum of the ε-form for the first time.

## Supporting Information Available

Example raw spectra, PCA scores versus time plots, representative spectra from turning points in the PCA score space, and loading plots from PLS-DA (PDF)
