# Supplementary material for: Low- versus Mid-frequency Raman Spectroscopy for in Situ Analysis of Crystallization in Slurries
Source: Mol Pharm. 2022 May 3;19(7):2316–26. doi: 10.1021/acs.molpharmaceut.2c00126 (PMC9257757; doi:10.1021/acs.molpharmaceut.2c00126)
Supplement: Supplementary file 1 — mp2c00126_si_001.pdf [file mp2c00126_si_001.pdf]

Electronic Supplementary Material for: Low- *versus* mid-frequency Raman spectroscopy for *in situ* analysis of crystallization in slurries

Jaana Koskela[1]; Joshua J. Sutton[2]; Tiina Lipiäinen[1]; Keith C. Gordon[2]; Clare J. Strachan[1]; Sara J. Fraser-Miller[2]*

[1]Drug Research Program, Division of Pharmaceutical Chemistry and Technology, Faculty of Pharmacy, University of Helsinki, Helsinki, Finland

[2]The Dodd-Walls Centre for Photonic and Quantum Technologies, Department of Chemistry, University of Otago, Dunedin, New Zealand.

List of supplementary figures

Figure S1 Example raw spectra collected each hour from a 25 ˚C in situ run. Note the low frequency domain has greater signal intensity than the mid-frequency counterpart. 3

Figure S2 Loadings versus time for the MFR data, (a) PC1 versus time, (b) PC2 versus time, and (c) PC3 versus time. Please note that the associated loadings are presented in Figure 3b of the manuscript...4

Figure S3 Loadings versus time for the LFR data, (a) PC1 versus time, (b) PC2 versus time and (c) PC3 versus time. Please note that the associated loadings are presented in Figure 4b of the manuscript...5

Figure S4 Average spectra from the turning points in PC space and the differences in these turning points in comparison to reference spectra for run A (a) and run B (b)..6

Figure S5 FT-IR PLS-DA weighted regression coefficients in comparison to amorphous and alpha forms of indomethacin. One factor was used for this analysis. 7

Figure S6 FT-Raman weighted regression coefficients in comparison to amorphous and alpha forms of indomethacin. One factor was used for this analysis. 7

Figure S7 In situ LF Raman PLS-DA weighted regression coefficients in comparison to amorphous and alpha forms of indomethacin. One factor was used for this analysis.......................................8

Figure S8 In situ MF Raman PLS-DA weighted regression coefficients in comparison to amorphous and alpha forms of indomethacin. One factor was used for this analysis.....................8

Figure S9 In situ LF + MF Raman PLS-DA weighted regression coefficients in comparison to amorphous and alpha forms of indomethacin. One factor was used for this analysis.....................9

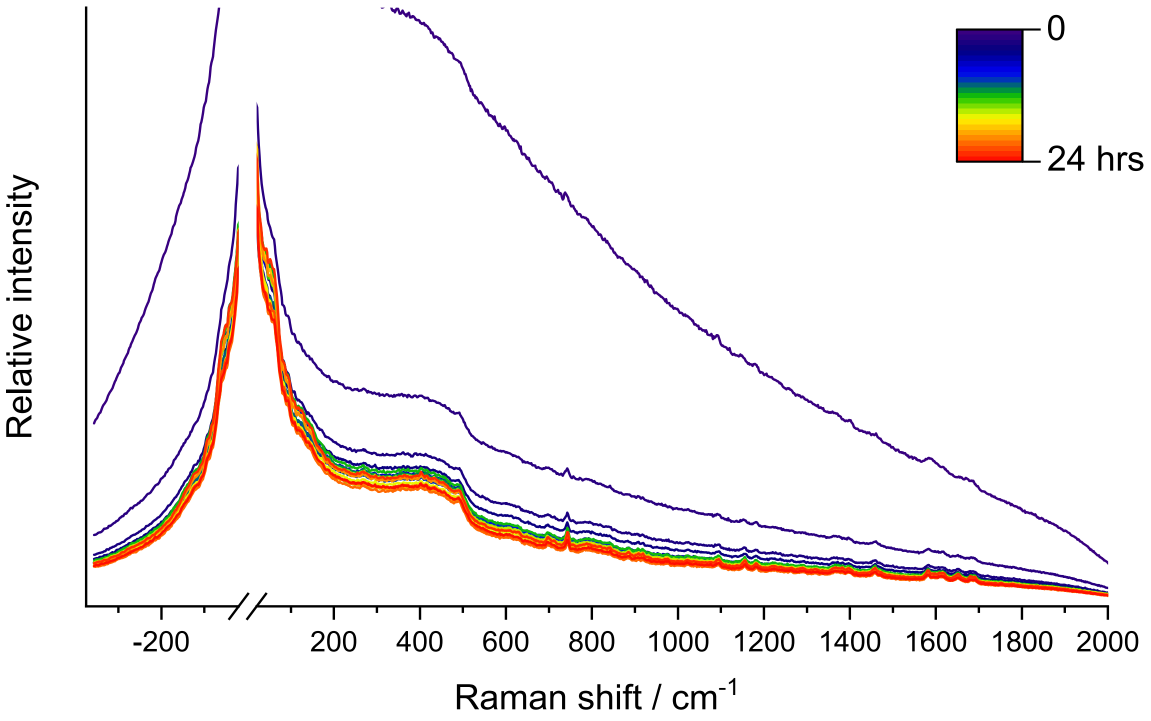

Figure S1 Example raw spectra collected each hour from a 25 ˚C in situ run. Note the low frequency domain has greater signal intensity than the mid-frequency counterpart.

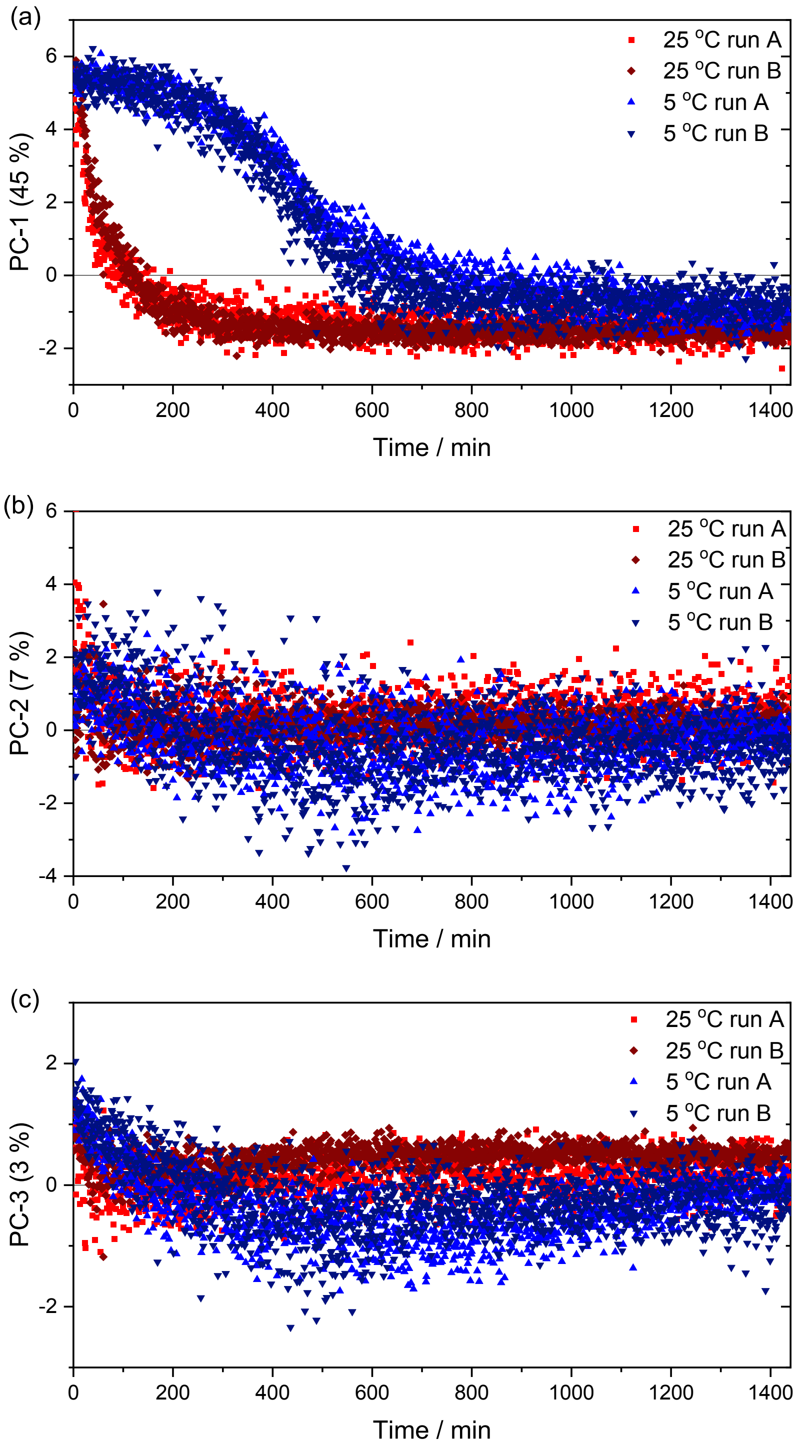
(a)
PC-1 (45 %)
Time / min
25 °C run A
25 °C run B
5 °C run A
5 °C run B
(b)
PC-2 (7 %)
Time / min
25 °C run A
25 °C run B
5 °C run A
5 °C run B
(c)
PC-3 (3 %)
Time / min
25 °C run A
25 °C run B
5 °C run A
5 °C run B

Figure S2 Loadings versus time for the MFR data, (a) PC1 versus time, (b) PC2 versus time, and (c) PC3 versus time. Please note that the associated loadings are presented in Figure 3b of the manuscript.

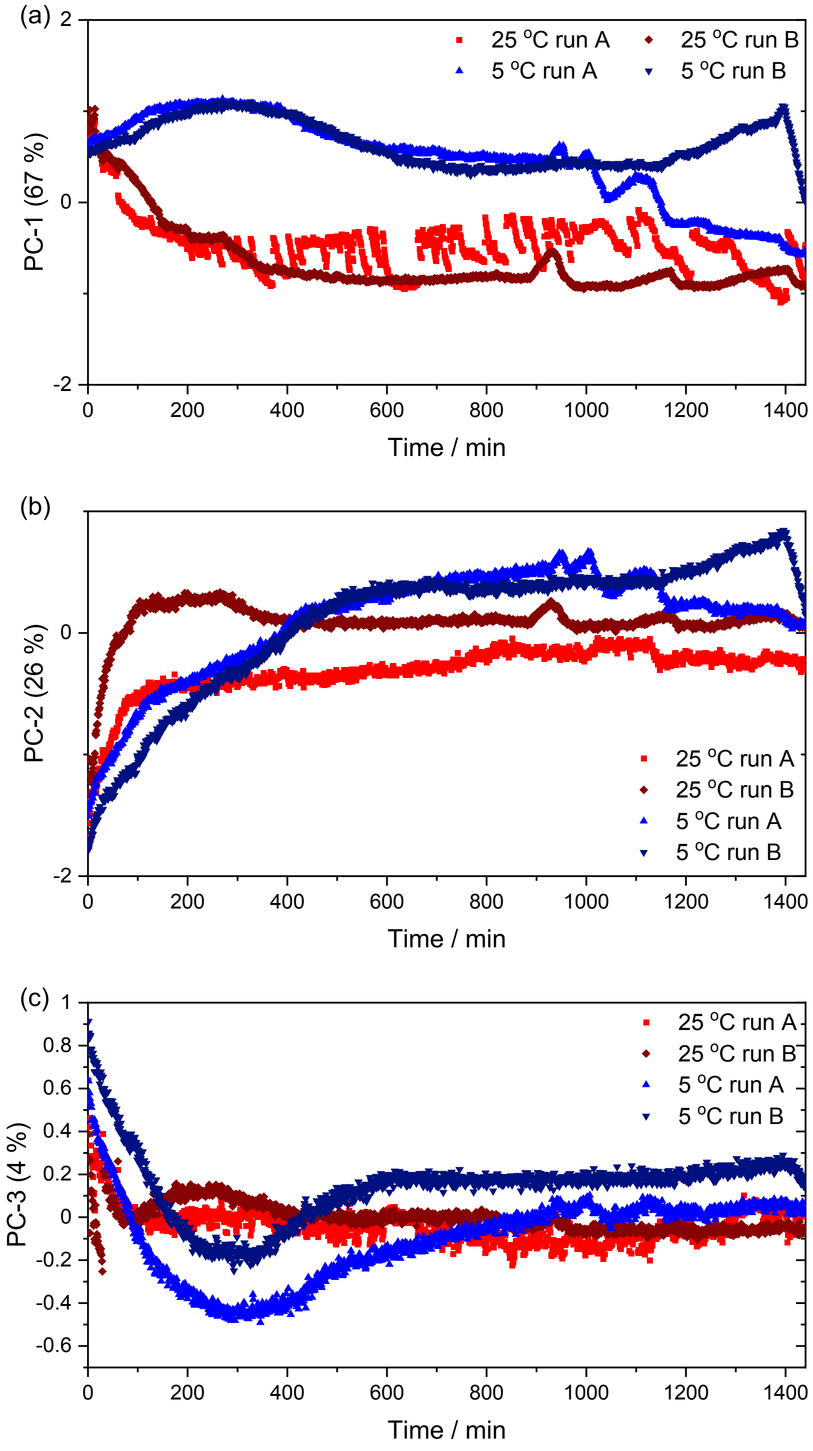
(a)
PC-1 (67 %)
Time / min
25 °C run A
25 °C run B
5 °C run A
5 °C run B
(b)
PC-2 (26 %)
Time / min
25 °C run A
25 °C run B
5 °C run A
5 °C run B
(c)
PC-3 (4 %)
Time / min
25 °C run A
25 °C run B
5 °C run A
5 °C run B

Figure S3 Loadings versus time for the LFR data, (a) PC1 versus time, (b) PC2 versus time and (c) PC3 versus time. Please note that the associated loadings are presented in Figure 4b of the manuscript.

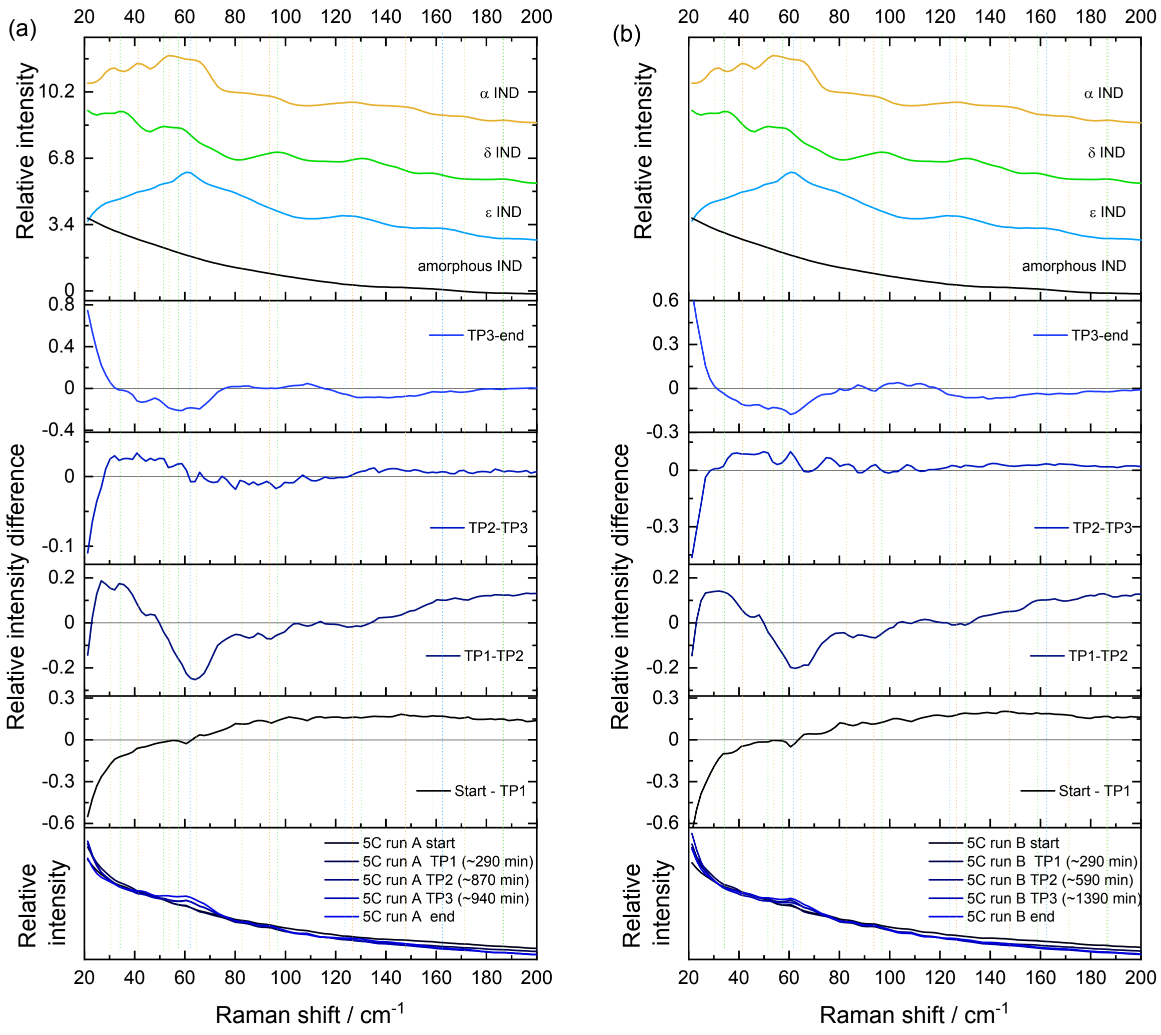

Figure S4 Average spectra from the turning points in PC space and the differences in these turning points in comparison to reference spectra for run A (a) and run B (b).

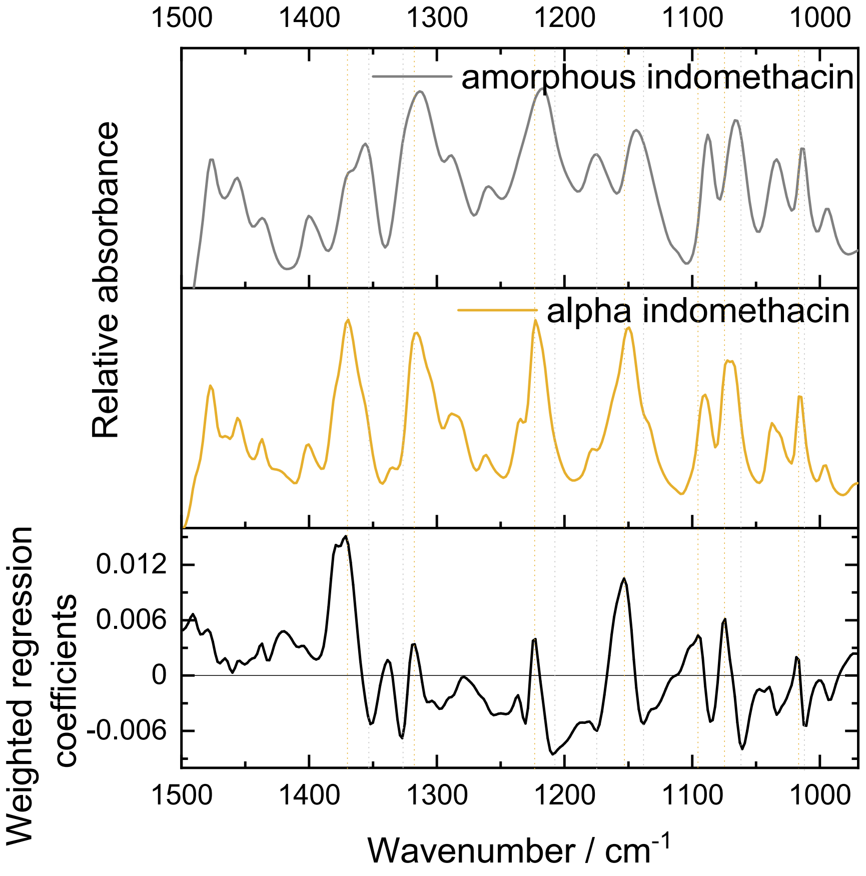

Figure S5 FT-IR PLS-DA weighted regression coefficients in comparison to amorphous and alpha forms of indomethacin. One factor was used for this analysis. The weighted regression coefficients are consistent with subtle differences in peak shapes between α- and amorphous indomethacin with negative regression coefficients attributed to amorphous and positive regression coefficient features more consistent with α-form features.

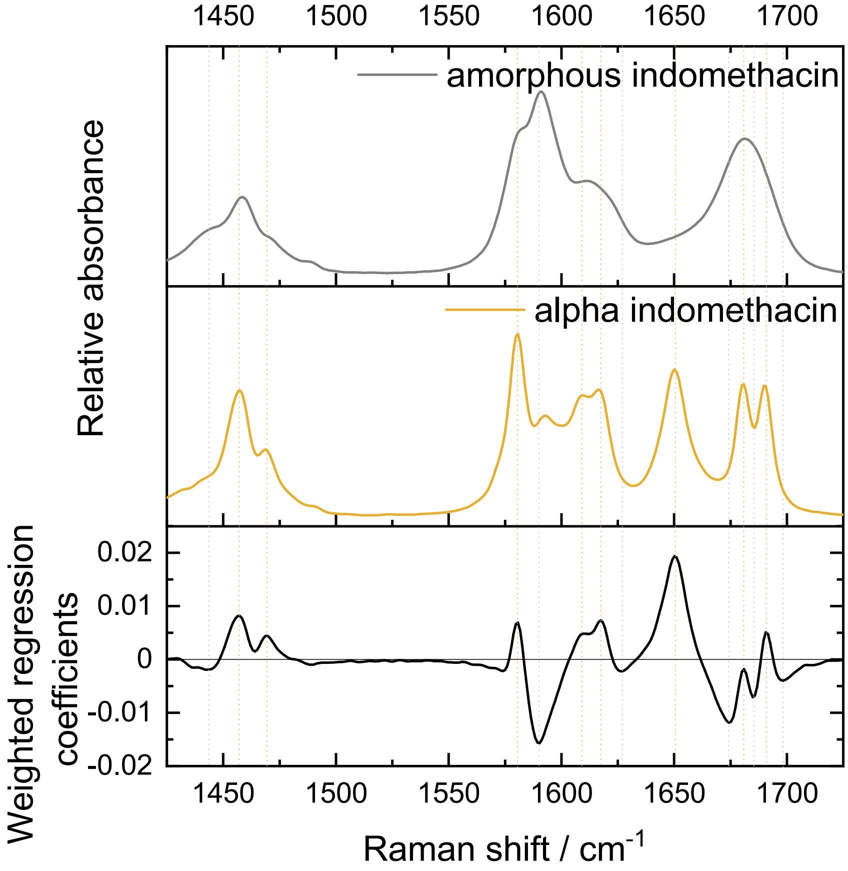

Figure S6 FT-Raman weighted regression coefficients in comparison to amorphous and alpha forms of indomethacin. One factor was used for this analysis. The weighted regression coefficients are consistent with the different peaks observed between α- and amorphous indomethacin with negative regression coefficients consistent with amorphous peaks and positive regression coefficient features consistent with α-form features.

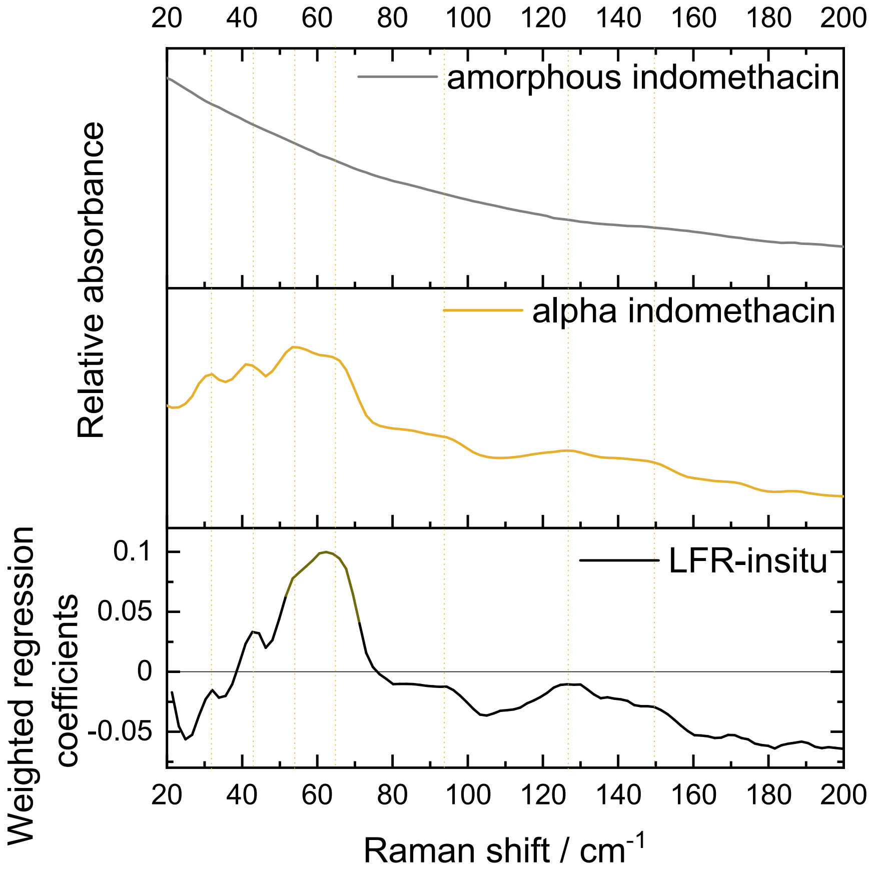

Figure S7 In situ LF Raman PLS-DA weighted regression coefficients in comparison to amorphous and α-forms of indomethacin. One factor was used for this analysis. The weighted regression coefficients are consistent with the difference between the amorphous VDOS (negative coefficients) and the α-form low frequency Raman spectrum (positive coefficients).

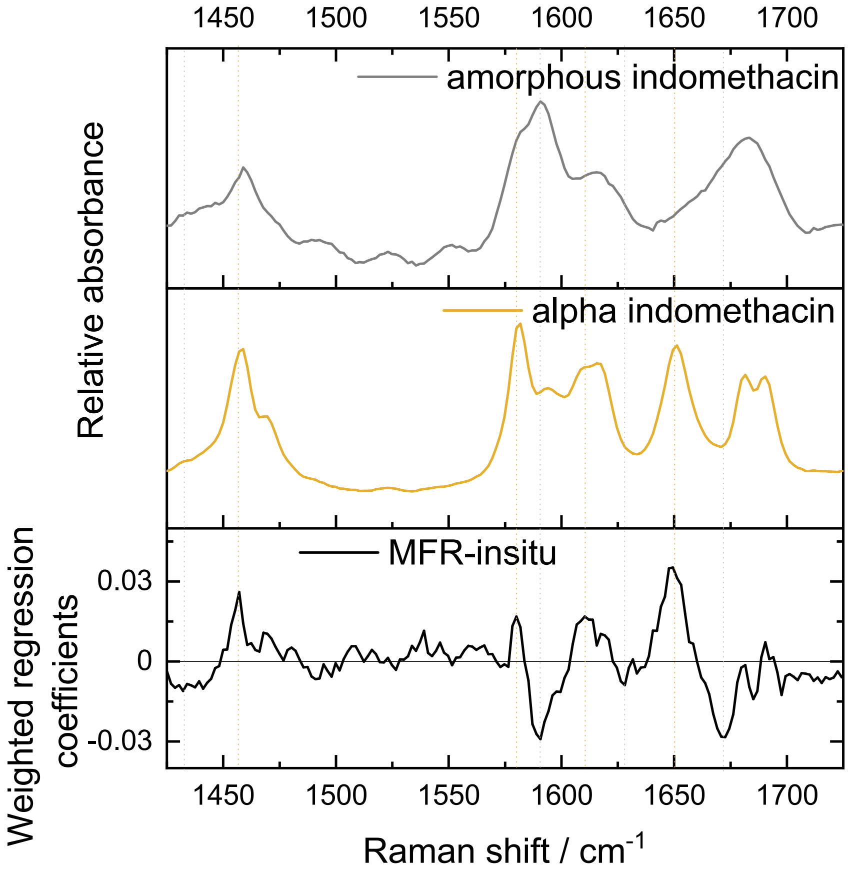

Figure S8 In situ MF Raman PLS-DA weighted regression coefficients in comparison to amorphous and α-forms of indomethacin. One factor was used for this analysis. The weighted regression coefficients are consistent with the different peaks observed between α- and amorphous indomethacin with negative regression coefficients consistent with amorphous peaks and positive regression coefficient features consistent with α-form features.

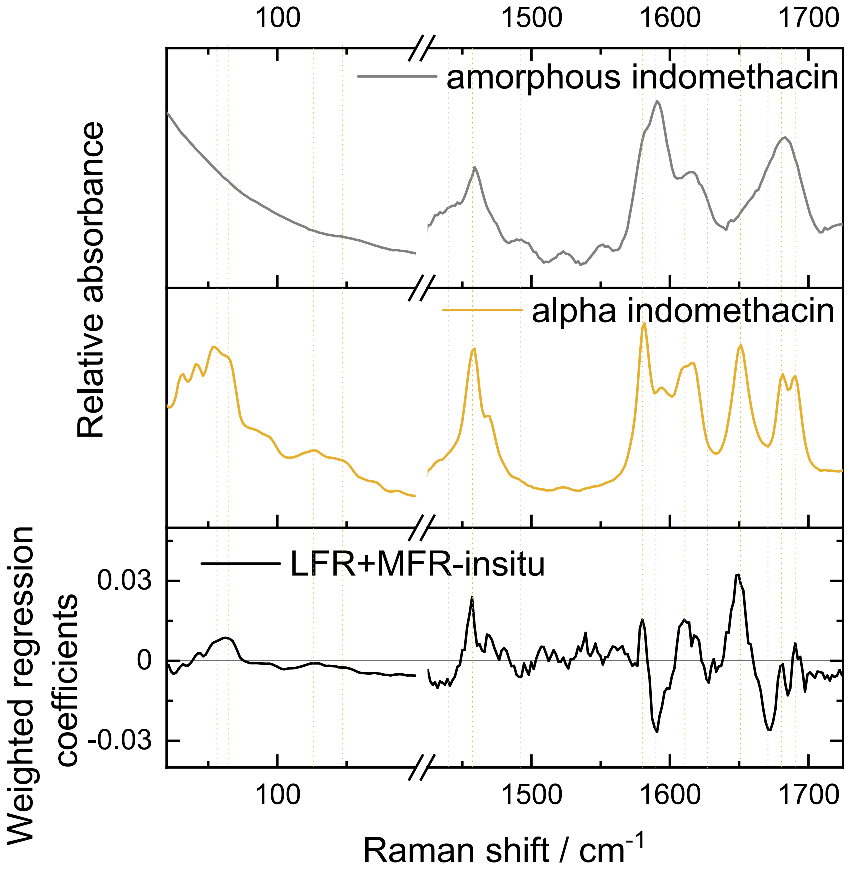

Figure S9 In situ LF + MF Raman PLS-DA weighted regression coefficients in comparison to amorphous and α-forms of indomethacin. One factor was used for this analysis. The weighted regression coefficients are consistent with the direct combination of those shown for the individual spectral regions (Figs S7 and S8) with the positive weighted regression coefficients being consistent with α-form and negative coefficients consistent with amorphous indomethacin.
